# Development of a decision tree diagram for classifying study designs in tumour pathology research: a multidisciplinary approach

**DOI:** 10.1002/2056-4538.70056

**Published:** 2025-11-29

**Authors:** Oana M Craciun, Ester García‐Ovejero, Fiona Campbell, Marina Montes‐Mota, Stefan Holdenrieder, Inga Trulson, Karolina Worf, Sophie Gabriel, Magdalena Kowalewska, Irmina Michalek, Kateryna Maslova, Lukasz Taraszkiewicz, Javier del Águila, Richard Colling, Puay Hoon Tan, Gabrielle Goldman‐Lévy, Christine Giesen, Ramon Cierco Jimenez, Dilani Lokuhetty, Ian A Cree, Iciar Indave, Beatriz Pérez Gómez, Magdalena Chechlińska, Marina Pollán Santamaría, Elena Plans‐Beriso, WCT EVI MAP Project Team, Nur Diyana Md Nasir, Michael Gilch, Clarissa Jing Wen Wong, Mervyn Ong, Joanna Didkowska, Zi Long Chow, Ruoyu Shi

**Affiliations:** ^1^ National Center for Epidemiology Instituto de Salud Carlos III Madrid Spain; ^2^ Doctoral School, PhD Program in Epidemiology and Public Health Universidad Autónoma de Madrid Madrid Spain; ^3^ Population Health Sciences Newcastle University Newcastle Upon Tyne UK; ^4^ Institute of Laboratory Medicine TUM University Hospital, German Heart Centre Munich Germany; ^5^ Department of Molecular and Translational Oncology Maria Sklodowska‐Curie National Research Institute of Oncology Warsaw Poland; ^6^ Department of Cancer Pathology Maria Sklodowska‐Curie National Research Institute of Oncology Warsaw Poland; ^7^ Department of Cancer Biology Maria Sklodowska‐Curie National Research Institute of Oncology Warsaw Poland; ^8^ Greater Poland Cancer Registry Greater Poland Cancer Centre Poznan Poland; ^9^ Nuffield Department of Surgical Sciences University of Oxford Oxford UK; ^10^ Department of Cellular Pathology Oxford University Hospitals NHS Foundation Trust Oxford UK; ^11^ Department of Pathology Luma Medical Centre Singapore; ^12^ International Agency for Research on Cancer (IARC) World Health Organization Lyon France; ^13^ Consortium for Biomedical Research in Epidemiology and Public Health (CIBERESP) Madrid Spain

**Keywords:** decision trees, neoplasms, epidemiologic study characteristics, review literature as topic, study characteristics, evidence‐based medicine, World Classification of Tumours, WCT, methods, WCT EVI MAP project

## Abstract

The World Health Organization (WHO) Classification of Tumours: A Living Evidence Gap Map by Tumour Type (WCT EVI MAP) project aims to develop Evidence Gap Maps of the available evidence, primarily to inform the WHO Classification of Tumours. The project, covering all tumour types, faces the challenge of reviewing a huge number of studies by reviewers from multiple backgrounds. The aim was to develop a decision tree (DT) diagram for classifying study designs reporting on tumour pathology studies, in order to support the decision‐making process when assigning evidence levels across various disciplines. A modified consensus process, incorporating stakeholder workshops, was conducted in three phases: (1) development of the initial DT diagram draft (literature review and expert evaluation); (2) iterative reviews with project partners; and (3) testing the advanced DT diagram version with several sets of references to refine critical points. A total of 368 records were used for training throughout the entire process. Consensus was achieved when classifications could categorise studies consistently without causing discordance in new example sets. A DT diagram and its Glossary of Operational Definitions with 27 decision nodes and 26 categories were developed. The DT diagram is organised into six sections: WCT EVI MAP selection criteria, evidence synthesis, basic research related studies, descriptive studies, observational and experimental studies, and diagnostic test studies. The DT diagram is a valuable tool for the project's needs, successfully integrating diverse disciplinary perspectives for classifying evidence in tumour pathology research according to study design. It lays the foundation for future advancements in evidence mapping and classification within tumour pathology and related disciplines.

## Introduction

The project ‘Mapping the Evidence for the World Health Organization (WHO) Classification of Tumours: A Living Evidence Gap Map by Tumour Type’ (WCT EVI MAP) is an international initiative aimed at synthesising the available evidence on tumour classification research to produce evidence gap maps (EGMs), for all currently defined tumour types, and to support decisions of the WHO Tumour Classification (WCT). This project is rooted in Evidence‐Based Medicine (EBM), which ensures a rigorous and comprehensive approach to integrating the best available evidence with clinical expertise to inform health decision‐making [1]. Furthermore, the WCT EVI MAP project seeks to advance the field and develop the concept and practice of Evidence‐Based Pathology (EBP), helping to establish this emerging practice within the broader evidence synthesis landscape [1, 2].

EGMs are a method of evidence synthesis that provides a visual summary of the available evidence on a given topic, to be summarised and visualised in an easy‐to‐interpret map format [3]. Adapted for tumour classification, the EGMs inform decision‐makers and allow the use of the best available evidence while minimising misinformation [4]. The proposed EGM for the WCT EVI MAP will be structured along two dimensions, as columns and rows: tumour type [1] and research domains [2], such as clinical characteristics, epidemiology, aetiology, pathogenesis, diagnostic features, prognosis, and prediction. Each cell in the EGM will contain a visual representation of the available evidence (e.g., published studies) for that tumour type and attribute. This is displayed as a number of bubbles of varying sizes and colours. The size of the bubble represents the number of studies contributing to that cell, and the colour represents the study design [5]. The colours also indicate the confidence the reader can have in those studies, based upon our ‘Hierarchy of Research Evidence for Tumour Pathology’, which has been adapted from traditional EBM levels of evidence [6]. EGM methodology does not involve the assessment of the quality of each study as in other evidence synthesis methods, an aspect that would inevitably require in‐depth reading of each article [3]. Therefore, accurately identifying the study design of a piece of evidence is a critical step in the EGM process.

Given the extensive scope of the WCT EVI MAP project, which spans all types of tumours, completing the evidence synthesis efficiently is a significant challenge. The multidisciplinary nature of this project further complicates the classification process, as the teams involved come from diverse backgrounds, such as pathology, biology, and epidemiology. Therefore, it was essential for us to establish a common ground in understanding different study designs and to create a tool that could be universally applied across disciplines. To date, various classification systems and flow charts for study designs have been proposed [7, 8, 9, 10, 11, 12]; however, these are often complex and inconsistent. This variety of approaches accounts for the conceptual complexity of the topic addressed. It was essential to reach an agreement between teams, for which it was necessary to employ an open consensus methodology to explore the priorities, ideas, and barriers among the different groups [13]. To this end, the development of a decision tool adapted to the needs of the WCT EVI MAP project, hereafter referred to as the decision tree (DT) diagram, was proposed. A DT diagram is a structured tool for decision‐making that helps establish a consistent classification system among reviewers. It operates by guiding the user through a series of decision nodes based on study attributes, ultimately leading to more subtrees or to a classification outcome [14]. Each instance – in this case, each study – is classified starting from a root node, progressing through several decision nodes, and ultimately guiding the reviewer to a specific design classification [14].

The objective of this study was to develop a DT diagram for classifying study designs derived from tumour pathology studies in order to support the decision‐making process when assigning evidence levels across disciplines.

## Materials and methods

The development of the DT diagram and the accompanying Glossary of Operational Definitions for the WCT EVI MAP project followed a structured, iterative methodology grounded in Consensus and Stakeholder Workshops [13]. With the availability of videoconferencing, the Consensus and Stakeholder Workshop method has been adapted to the online environment to reach all project partners. Consensus was defined as being reached when the users reliably classified studies using the DT diagram across multiple references from the scientific literature.

**Figure 1:**
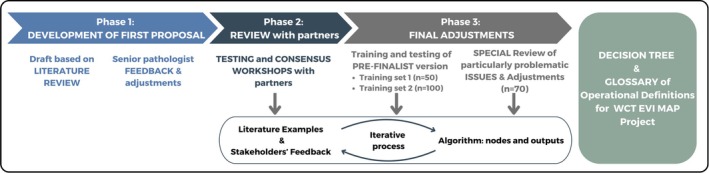
Summarised development process of the decision tree diagram and glossary of operational definitions.

Between June 2023 and February 2024, we conducted a three‐phase process, using moderated sessions and iterative feedback, to create a robust tool for classifying study designs (see Figure 1).

### Phase 1: development of the first proposal for a DT diagram and glossary of operational definitions

The initial phase of development was led by a moderating team of five expert epidemiologists (*n* = 5) and an evidence synthesis specialist. This team identified several issues and discordances in knowledge, concepts, and difficulties in classifying study designs among all the project partners. A first draft of the DT diagram was developed by this group based on available literature, such as other published DTs and classifications [7, 8, 9, 10, 11, 12]. It incorporated the categories previously collected in a pathologist‐led study [6].

This first draft was circulated among the leading WCT EVI MAP expert researchers (*n* = 3) from various fields, including expert pathologists, expert molecular biologists, and senior epidemiologists, to ensure clarity and comprehensibility of both language and structure. Based on expert feedback, we revised the branch order and clarified specific decision nodes. Significant adaptations were required to address unique aspects of tumour pathology and the specific needs of the WCT EVI MAP project.

A Glossary of Operational Definitions (henceforth referred to as Glossary) of each of the categories and decision nodes appearing in the DT diagram was drafted with a practical focus to complement and assist in its use [1, 7, 8, 11, 12, 15, 16, 17, 18, 19, 20, 21, 22, 23, 24, 25, 26]. Due to the workload of the project, the decision nodes were designed to work with the information contained in the study abstracts.

### Phase 2: iterative review with partners

The second phase involved iterative review and refinement through a series of workshops, organised and led by the moderating team. The participants comprised project members (*n* = 14) representing the main stakeholders who would use the tool (pathologists, molecular biologists, epidemiologists, PhD students). The International Agency for Research on Cancer (IARC) and WCT EVI MAP project teams provided a set of 168 tumour‐related references, which were used as test sets and selected to cover all relevant study designs for tumour pathology.

Consensus Workshops were held through online videoconferencing where the moderating team coordinated small breakout groups and then shared the results, concerns, and issues that had arisen with all participants. Feedback on problems or issues related to the classification of these studies was received by the moderator team during the online workshops and was also submitted from the different project teams *via* email. All feedback received was compiled, and the decision nodes, order, and outcomes of the DT diagram were iteratively adjusted after each workshop. If participants identified a specific problem, they could provide examples of references to the moderator team to be added to the subsequent set of references for the next online workshop. This process was repeated up to five times until a pre‐final DT diagram was agreed upon.

During the development of the DT diagram, attention was paid to ensuring that the categories and questions were clear, sufficient, coherent, relevant, and pertinent, looking to improve ease of application and consistent classification outcomes.

To enhance the usability, virtual versions of the DT diagram and Glossary were developed using Genially®, enabling interactive navigation of nodes and definitions [27].

### Phase 3: final adjustments

The final phase focused on fine‐tuning the pre‐final DT diagram through comprehensive training and feedback in two final consecutive online workshops. Two training sets, consisting of 50 and 100 references, respectively, were used to support the development of the decision tool and to explore its practical use among the 14 participants.

Examination of the responses to these training sets, and of the comments from the participants at this stage, identified four critical nodes or categories where there was a high level of disagreement. Targeted reference packages were prepared for each problematic area: clinical guidelines, consensus studies, and reviews (*n* = 25); human data definition (*n* = 10); diagnostic tests branch (*n* = 15); and case control versus cross‐sectional node (*n* = 20). Further adjustments were made through collaborative discussions and email consultations with project teams to fine‐tune the final decision nodes and definitions.

## Results

**Figure 2:**
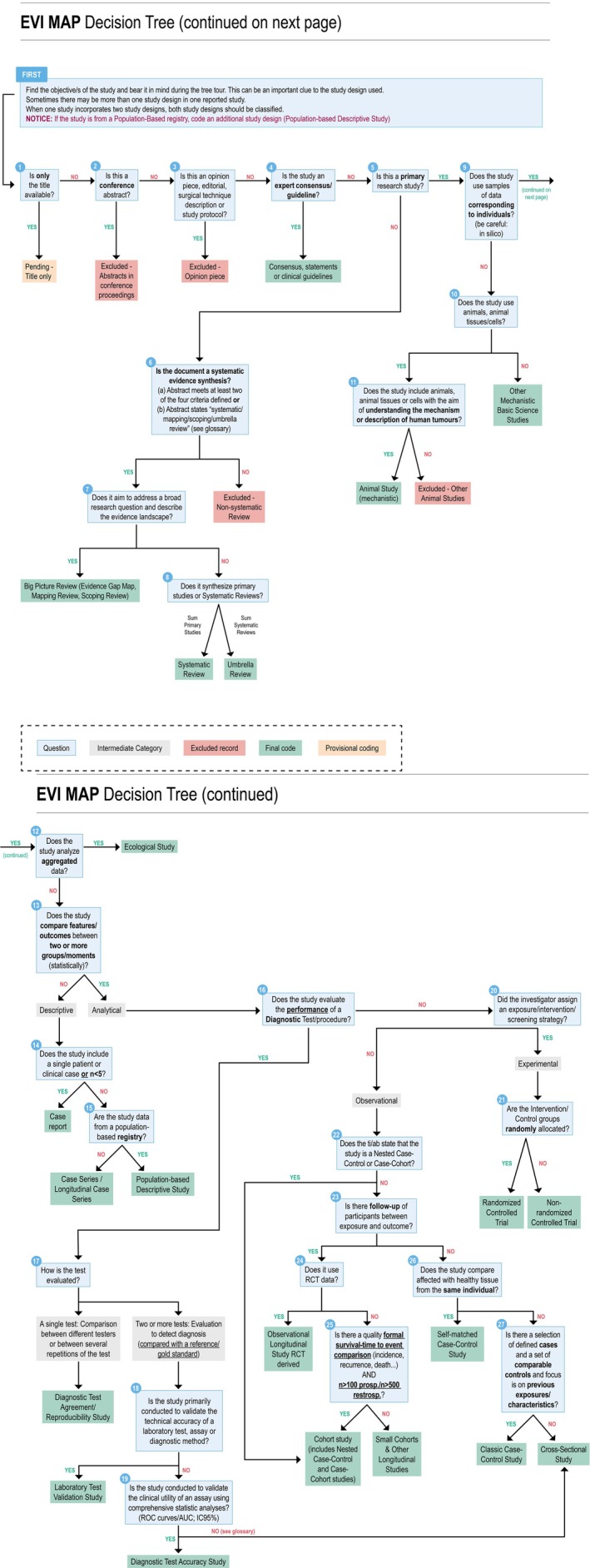
Decision tree diagram.

The DT diagram (Figure 2) and the Glossary (supplementary material, File S1) resulted in the formulation of 27 decision nodes and 26 categories. The DT diagram can be structured into six key sections, as follows: (1) WCT EVI MAP selection criteria, (2) evidence synthesis, (3) basic research‐related studies, (4) descriptive studies, (5) observational and experimental studies, and (6) diagnostic test research studies (Table 1).

**Table 1:** Sections of the DT diagram and summary of the included categories in each section

Decision tree diagram sections	Containing categories and study designs
Project's selection criteria	Title Only (pending); Abstracts in conference proceedings (excluded); Opinion piece (excluded)
Evidence synthesis	Consensus, statements, or clinical guidelines; big picture review; Systematic Review; Umbrella Review; Non‐Systematic Review (excluded)
Basic research‐related studies	Animal Study (mechanistic); Other Mechanistic Basic Science Studies; Other Animal Studies (excluded)
Descriptive studies	Case Report; Case Series/Longitudinal Case Series; Population‐Based Descriptive Study
Other observational and experimental studies	Ecological Studies; Randomised Controlled Trial (RCT); Non‐Randomised Controlled Trial (Non‐RCT); Observational Longitudinal Study RCT derived; Self‐Matched Case–Control Study; Classic Case–Control Study; Cross‐Sectional Study; Cohort Study; Small Cohorts & Other longitudinal Studies
Diagnostic test research studies	Diagnostic Test Agreement/Reproducibility Studies; Laboratory Test Validation Studies; Diagnostic Test Accuracy Studies

While the majority of the study designs included in the DT diagram are well‐established and defined in the existing literature, certain modifications and adaptations were introduced to suit the specific objectives of the WCT EVI MAP project. These tailored modifications are discussed below, highlighting both methodological and conceptual modifications.

The first part of the DT diagram (decision nodes 1–3) is dedicated to selection criteria, which were necessary for the application of the DT diagram during the study selection process in the reviews that will be made within the project. These criteria ensured that the studies included in the framework aligned with the overarching goals of the project.

Decision node 4 asks, ‘Is the study an expert consensus/guideline?’ Although this refers to documents that differ greatly in their methodological approaches, distinguishing between them based solely on abstract‐level information proved challenging. Consequently, these were merged into a single category.

To meet the need to approximate the quality of the studies in order to classify them into different levels of evidence within the EGM domains, not only was the design considered but other study characteristics – such as data source – were also considered. Thus, a specific reference to population‐based studies was included in Node 15 as they are the most suitable for epidemiological description (domain included in the project's framework). Additionally, Node 24 emphasises observational studies derived from randomised controlled trials (RCTs), which are often classified as high quality due to their robust data sources, as highlighted by pathologists.

Node 13 addresses the role of statistical analysis in determining the analytical nature of the study. While statistical analysis does not *per se* define the analytical character of an investigation, this subdivision allowed for an assessment of the depth of comparisons. Thus, in the category ‘Case series/Longitudinal case series’, cross‐sectional studies with less developed analysis are included, and included in the category ‘Small Cohorts & Other Longitudinal Studies’ are prospective case series with analyses that make them capable of more elaborate conclusions. On the other hand, the category ‘Small Cohorts & Other Longitudinal Studies’ was created to distinguish these studies from cohorts characterised by complex analyses suggesting a more careful development, prospective data collection reflecting their higher quality, or larger sample sizes and better control of follow‐up losses, which altogether can provide more precise estimates.

The studies included in the diagnostic test branch (nodes 16–19) are based on ‘cross‐sectional studies’ or ‘case–control studies’ and they could be classified with that nomenclature. The reason for creating the diagnostic test branch was to be able to specifically identify those studies that evaluated ‘diagnostic accuracy’ and ‘reproducibility’ inside the WCT EVI MAP framework. In that sense, only those that refer to the accuracy or precision of a medical diagnosis or diagnostic test will follow this branch, while those concerning staging or prognosis will be directed towards other design categories.

For a better adjustment in the levels of evidence designed for the future EGMs of the project, several study designs are grouped under broad labels. For example, the ‘Case Reports’ category also encompasses case series with fewer than five cases. Additionally, some cross‐sectional studies lacking statistical analysis are categorised as ‘Case Series’ due to their lesser depth of analysis.

Given the complexity and nuances implemented by these modifications, the Glossary (supplementary material, File S1) provides a guide to navigating the DT, with category definitions, clarifications, and tips to ensure consistent classification.

## Discussion

The development of the DT diagram represents a significant advancement in the classification of study designs within the field of tumour pathology, addressing a long‐standing gap in evidence synthesis that we identified. As the first tool specifically designed for this purpose in tumour pathology, it enables a systematic categorisation of study designs that often defy traditional classifications. Pathology‐related studies, with their diverse methodologies and evolving research approaches, present unique challenges in the clear delineation of study designs. This tool bridges gaps and addresses the complexities and nuances inherent in tumour pathology research, which we believe allows for more consistent evidence mapping.

### Consensus method: process dynamics and transdisciplinary collaboration

Consensus methods have broad applications across various research domains and facilitate the creation of agreements in a transparent, inclusive, and rigorous manner [13]. These methods have been successfully applied to diagnostic algorithms [28], nomenclature [29], education, and research prioritisation [30], and other contexts. Although other consensus methods, such as Delphi and nominal group techniques, are commonly used, these rely on rounds of voting and a more structured approach to establish categories [12]. We felt we needed a more flexible qualitative approach for such a complex problem. In our study, we adopted a consensus‐driven, collaborative, and learning‐oriented process.

An essential aspect of our method was the iterative and controlled feedback loop, facilitated by a moderating and coordinating group that employed multiple feedback mechanisms: consensus meetings in large and small groups, open comments within training questionnaires, and email communication when needed. This approach allowed us to engage with experts from different fields, and it led to a deeper understanding of the perspectives, concepts, and terminologies used in each discipline. Due to the nature and purpose of the WCT EVI MAP project, the development of the DT diagram was carried out using a transdisciplinary approach that has been well documented in the literature [31, 32] as essential for co‐producing a tool that could be broadly used across different research domains. In addition to developing the tool for guiding the classification of study designs, the process itself fostered the formation of a cohesive team—an added benefit of the method. Effective inter‐team communication and decision‐making procedures were critical for the success of the DT diagram development. The challenges encountered, the collaborative processes, and the benefits derived from our approach align with what has been described in other studies in which it has been shown to be a necessary approach for the creation of new and extensive knowledge [32, 33, 34, 35]. This sense of cohesion and collaborative capacity will ultimately contribute to addressing future challenges within the WCT EVI MAP project.

### From the initial idea to a tool with a future

The final tool is less parsimonious than initially envisioned. Also, some of the final categories included in the DT diagram are not conventional study designs, especially those concerning the analysis method or data source. Each DT diagram node, final category, and clarification added to the Glossary was the result of consensus processes, and this was necessary for the DT diagram to serve its purpose.

The inclusion of multiple clarifications in the Glossary reflects the need for a tool that accurately addresses the complexities of classifying study design. Due to time constraints that prevent us from being able to review the full text of each study for the EGMs, some of these decisions were necessary to make the tool applicable *via* abstracts and therefore are pragmatic within the framework of the WCT EVI MAP project.

Furthermore, the DT diagram developed in this study is not only an effective tool for our project but also has potential for broader applications. It could be particularly useful in future efforts aimed at semi‐automated classification of studies, especially with the integration of machine learning techniques. The detailed, machine‐readable description of the categories given in the DT and Glossary could serve this purpose. In addition, while it would be necessary to adapt it for full text assessment, there is clear practical utility in abstract screening, a step common to all evidence synthesis methodologies.

### Strengths and limitations

This study's key strength lies in its innovative approach to the classification of study designs in tumour pathology‐related research, an area marked by blurred boundaries and inconsistent terminology. The collaborative, transdisciplinary, and iterative process behind the development of the DT diagram allowed us to include many different insights and enhance its relevance and applicability. Although we have made extensive efforts to capture a wide range of study designs, the broad application of the DT diagram may reveal some limitations or areas requiring adjustments. Therefore, it is important to remain open to refining the tool as it is tested in various contexts. Furthermore, we intend to conduct a quantitative study designed to investigate inter‐rater agreement in study classification using the DT diagram, with the aim of evaluating its validity and reliability.

This study introduces a pioneering tool for classifying study designs in tumour pathology‐related research, addressing a critical gap where traditional study categorisations often fail to account for the complexities of the field. It lays the foundation for future advancements in evidence mapping and classification within tumour pathology and related disciplines.

## Author contributions statement

MC and FC contributed to the original idea of adapting existing study design decision diagrams to the WCT EVI MAP objectives. FC developed the initial draft of the DT diagram, providing a starting point that served as the basis for its development. The team consisting of OMC, EG‐O, BPG, MPS, and EP‐B was responsible for expanding, developing, and refining the primary idea, applying their expertise in epidemiology; they led the development and designed the study methodology and formed the moderation team for the consensus meetings and analysis of feedback responses. OMC, EG‐O, SH, MC, FC, BPG, MPS, and EP‐B contributed to the overall conceptualisation. OMC, EG‐O, SH, IT, KW, SG, MC, MK, IM, KM, LT, JdA, RC, PHT, GG‐L, CG, RC, DL, IAC, FC, BPG, MPS and EP‐B participated in the research, development and final refinement of the DT diagram. OMC, EG‐O and MM‐M wrote the first draft of the manuscript. All authors contributed to the critical revision of the manuscript. SH, MC, RC, PHT, DL, IAC, II, FC and MPS obtained financial support for the project that led to this publication.

## Supporting information


**File S1.** Glossary of operational definitions

## Data Availability

Data sharing is not applicable to this article as no datasets were generated or analysed during the current study.
