# Supplementary material for: Development of a decision tree diagram for classifying study designs in tumour pathology research: a multidisciplinary approach
Source: J Pathol Clin Res. 2025 Nov 29;12(1):e70056. doi: 10.1002/2056-4538.70056 (PMC12664524; doi:10.1002/2056-4538.70056)
Supplement: Supplementary file 1 — File S1. Glossary of operational definitions [file CJP2-12-e70056-s001.pdf]

**Development of a decision tree diagram for classifying study designs in tumour pathology research: a multidisciplinary approach**

OM Craciun, E García-Ovejero *et al. J Pathol Clin Res* https://doi.org/10.1002/2056-4538.70056

**Supplementary File S1**

## File S1. Glossary of operational definitions

# BEFORE STARTING

1. Find the objective/s of the study and bear it in mind during the tree tour. This can be an important clue to the study design used.
    a. It can be explicit in in the title.
    b. More frequently, it is stated in the first sentences of the abstract.
    c. If there is no objective: consider the main result as the main objective.
2. Sometimes there may be more than one study design in one reported study.
3. When one study incorporates two study designs, both study designs should be classified.
4. The objective/s should tell you to what part of the framework this paper contributes to (can be several).

*Agreement: **The study design stated by authors is accepted only if:** "nested case-control" "case-cohort" "RCT" "Non-RCT" or "Systematic/Scoping/Umbrella Reviews" The rest must be checked against our agreed criteria.*

**NOTICE: If the study is from a Population-Based registry, code an additional study design (Population-based Descriptive Study)** If the study is population-based, but does not derive from a population registry, the descriptive design should not be added. Hospital registries are not population-based. Molecular databases are not always Population – Based.

**Question 1: "Is only the title available?**

**Yes - Code: Pending - Title only:** Where an abstract is missing, but the title suggests the study should be included, code as pending.

**Question 2: "Is this a conference abstract?"**
The aim here is to exclude conference abstracts (for posters or oral presentations).

**Yes - Code: Excluded - Abstracts in conference proceedings.**

**Question 3: "Is this an opinion piece, editorial, surgical technique description or study protocol?"**

**Yes - Code: Excluded - Opinion piece.** The study is an opinion piece, an editorial, a letter to the editor or correspondence, a surgical technique description or a study protocol.

*Agreement: By abstract, sometimes it is not clear whether the study is an opinion article or a non-systematic review. If they do not clearly indicate that it is a review, they will be excluded as an opinion piece*.

**Question 4: "Is the study an expert consensus/guideline?"**

**Yes - Code: Consensus, statements, or clinical guidelines**.

***Consensus Study:*** An original study which seeks a consensus among experts using a formal methodology such as a Delphi study, an online or in person survey or voting process (consensus conference) and published as a position statement/recommendation. This is distinct from simple published surveys, letters to editors or opinion articles.

***Clinical Practice Guidelines*** are defined as systematically developed statements or processes to assist clinician and patient decisions about appropriate health care for specific clinical circumstances. We will include if the abstract states 'guideline or statement' developed by a panel of experts or professional societies or official institutions or its abstract refers to the use of some systematic methods for the development of clinical practice guidelines or recommendations (i.e. GRADE, IOM Standards, G-I-N Standards or Guidelines 2.0.

**Question 5: "Is this a primary research study?"** Directly gathering and analysing new data. This is distinct from secondary research (evidence synthesis) which gathers and analyses existing (usually published) data from prior studies.
*False friend "pooled":* In the context of systematic review + meta-analysis "pooled estimators" or "pooled analysis" are used to combine the results/estimators of different studies (it is a synthesis study). Outside the context of systematic review + meta-analysis, "pooled analysis" refers to the analysis of individual data of participants gathered from several studies (it is considered an original study).
*Clues for no: Words that indicate that there is synthesis of evidence: random effects model, I2, heterogeneity*

**No - Go to Question 6**
**Yes - Go to Question 9**

# SECONDARY RESEARCH

**Question 6: "Is the document a systematic evidence synthesis?**

a) Abstract states "systematic/ mapping/ scoping/ umbrella Review" or,
b) Abstract meets at least two of the four criteria defined.
   1. Structured research question (generally "PICOs") and Systematic search.
   2. Systematic presentation of findings: Flowchart showing selection process and summary of findings of included studies.
   3. Describe the method of synthesis.
   4. Risk of bias appraisal of each study.

*False friend, be careful: Meta-analysis: Sometimes meta-analysis is performed without the basis of a systematic review. In that case it would be excluded.*
*Clues for no: narrative synthesis*

**No - Code: Excluded - Non-systematic Review.**

**Question 7: "Does it aim to address a broad research question and describe the evidence landscape?**

**Yes - Code: Big Picture Review:** Systematic reviews which do not include a synthesis but seek to describe the evidence landscape. Includes: Mapping Reviews, Scoping Reviews and Evidence Gap Maps.

**Question 8: "Does it synthesize primary studies or Systematic Reviews?**

**Yes, it summarizes primary studies - Code: Systematic Review:** A review of the scientific evidence which applies strategies that limit bias in the assembly, critical appraisal, and synthesis of all relevant studies on the specific topic. With Meta-analysis: Systematic review followed by a statistical analysis of results from separate studies, examining sources of differences in results among studies, and leading to a quantitative summary of the results if the results are judged sufficiently similar or consistent to support such synthesis.

**Yes, it summarizes systematic reviews - Code: Umbrella Review:** Umbrella Reviews or Overviews of Reviews are reviews of systematic reviews. Overviews are often broader in scope than any individual systematic review, meaning that they can examine a broad range of evidence.

# PRIMARY RESEARCH

**Question 9: "Does the study use samples or data corresponding to individuals?"**
(i.e. "ten serrated polyps and ten polyps of another type" or "ten nodules of cirrhosis and ten of HCC" these are classified as data corresponding to individuals)
Human individual data may include molecular or genetic data. Cohort consortia or case controls included in repositories or databases (e.g. TGCA, practical, dbGaP), go downwards, **unless they only use the information for in silico analysis**.
If the only study design is an in-silico method, then it goes to basic science branch.

*False friend: 'in silico' Sometimes, "in silico" are an added analysis, as a way to enrich other types of designs. If so, the epidemiological design previously carried out must be codified.*
*Clues for no: 'xenograft models"*

**No - Go to Question 10**
**Yes - Go to Question 12**

**Question 10: "Does the study use animals, animal tissues/cells?"**
If the abstract does not specify that the data is from animals ("animal", "canine", "mice" or similar) it will be assumed that it is human data.

**No - Code**: **Other mechanistic basic science studies:** studies **without clinical data associated.** In vitro/in *silico* studies that use human derived samples **for fundamental/basic science/biological experimental work** (such as living cells lines or tissues, molecules, or studies using organoids, three-dimensional miniature versions of organs or tissues generated from cells with stem potential). This does not include studies of clinical tests carried out in the lab on patient tissues taken for clinical purposes (these are considered in vivo clinical/epidemiological or diagnostic studies instead). Are included here:

- **In silico studies:** computer simulation or evaluations of synthetic data with known properties.
- **Molecular database derived studies:** Databases from cohort or case-control consortia are considered human data (i.e., Practical, TGCA). The rest of studies using data derived from online molecular, genetic, or another biological database repository would be classified are included here.

**Question 11: "Does the study include animals, animal tissues or cells with the aim of understanding the mechanism or description of human tumours?"**
*Be careful: Studies on therapies or treatments (i.e. Murine antibodies) are not included.*

**Yes - Code: Animal Study:** A study that uses animals or animal samples (including cell lines) as research subject including xenograft models (models of cancer where the human tissue or cells are implanted into an animal). Studies carried out in the areas of basic research (mechanistic studies) and medicine referring to human oncology. Research concerning veterinary medicine purposes are to be excluded.

**No - Code: Excluded – Other Animal Studies**

**Question 12: "Does the study analyse aggregated data?"**

**Yes - Code: Ecological Study:** A study in which the units of analysis are populations or groups of people rather than individuals (**aggregated data**). Geographical comparisons with aggregated outcomes such as temporal trends including joint point analysis, time series, interrupted time series and correlations between aggregated variables.

*A further study design will be coded if the study **compares*** incidence/survival/mortality/recurrence **rates** across categories of an individual variable. This approach is common in articles that use population-based cancer registries as a data source.*
**See below (Question 13)*

**Question 13: "Does the study compare features/outcomes between two or more groups/moments (statistically)?"**
The research question seeks an association between one or more exposures/biomarkers/characteristics and the features/outcome of interest.

- **Considered groups**: Formal association or statistical analysis between groups with/without intervention or treatment, exposure/not exposure, anatomopathological characteristics, clinical features, molecular features, different tumour type tissues or individuals, healthy/non-healthy tissues, or individuals, two different moments in time. (i.e., p-value, regression, or other statistical tests).
  - **For diagnostic performance** (sensitivity, specificity, agreement between testers or similar**), percentages are considered as a valid measure as comparison.**
  - For non-RCT if it says in the title or abstract that the study is a "non-RCT", it would be coded as such.

  ***Agreed keywords accepted as comparison in abstract:** "association" (beware: it's not analytic when the verb "to associate" can be replaced by "to have coincidental" or "to have concurrent", i.e., PMID: 27494340); "correlation", "significantly"; "xxx analysis" (referred to statistical analysis i.e., Cluster analysis or Logistic analysis, principal component analysis); Predictive performance comparison; Quantifications or the probability of an event occurring (i.e., "twice probability"). Wouldn't work if it just mentions the frequency.*
  *Sometimes the study has a descriptive objective, but it exceeds this comparison criterion. If this is the case, the tree must be followed by the analytical branch.*
- **Not considered groups:** Percentages, descriptions, or trends by age, sex, ethnic groups, geographical regions etc, the goal is more descriptive without trying to test a hypothesis.

**No - Go to Descriptive studies.**
**Yes - Go to Analytical Studies.**

# DESCRIPTIVE STUDIES

**Question 14: "Does the study include a single patient or clinical case or less than five subjects?"**

**Yes - Code: Case Report:** Detailed description of observations at the individual level, documented from a **single** patient or clinical case. This code will also include case series with less than five subjects (n<5).

**Question 15: "Are the study data from a population-based registry?"**

**Note: Hospital registries are excluded here** – these are not population-based. The focus here is population-based registries which collect information on all new cancer cases that occur in a well-defined population, corresponding to a specific geographic area. The collection is done in a systematic way from several sources (usually following the IARC quality control criteria).

**No - Code: Case Series/ Longitudinal Case Series:** Includes several types of descriptive studies:

- Case series: A collection of subjects (usually patients) with common characteristics used to describe some clinical, pathophysiological, or operational aspect of a disease, treatment, exposure, or diagnostic procedure. Without non-tumoral comparison group
- Longitudinal case series: A single-group research design in which measurements are made at several different times, thereby allowing trends to be detected. **Note: Many studies use the term time series when using aggregated data (code as Ecological studies).**

*Clarification: For this classification tree, this category includes cross-sectional studies that do not meet the statistical comparison criteria defined in question 13.*

**Yes - Code: Population-based Descriptive Study:** Studies carried out from Population-based Cancer **Registries** (or other population-based registries) that seek to describe a population group, but without a comparison group (there may be comparisons based on sociodemographic variables such as sex, age, or ethnic groups). A hospital registry is not a population-based registry.

# ANALYTICAL STUDIES

**Question 16: "Does the study evaluate the performance of a diagnostic test or procedure?"**

This section includes studies assessing the performance of a diagnostic test, that is: **its reproducibility** (agreement between the test taken at different times, agreement between observers, Cronbach alpha, Cohen kappa, etc) and **studies comparing a test with another one considered the gold-standard** to assess the new test accuracy: ROC curve, AUC, sensitivity, specificity, predictive positive value (PPV) and predictive negative value (PNV) or comparison between positive and negative samples.

***Be careful: The evaluation performance of diagnostic test does not include the evaluation of screening strategies*** *(screening strategies usually compare the application of different diagnostic tools, strategies or procedures normally using a randomized clinical trial -randomly applying the diagnostic procedure/strategy/tool- and compare the results after follow-up. They are included as Randomized Clinical Trial or in the corresponding design if they are not randomized.*

***Be careful: The evaluation performance of diagnostic test does not include the evaluation performance of prognostic groups.*** *False friends: ROC CURVES (Area under curve (AUC), sensitivity…) They could be for Diagnostic Test but also for define prognostic groups.*

***To calculate the precision of a test (sensitivity, specificity, etc.),*** *the samples/patients* ***must be previously classified by another test (reference or "Gold standard"*** *- if it is considered the best method available -). Even if it is not referenced in the abstract, its existence can be deduced (there cases and non-cases or different types of cases) if those calculations are shown.*

**Yes - Go to Question 17**
**No - Go to Question 20**

**Question 17: "How is the test evaluated?"**

*False friend "agreement": sometimes is referred to accuracy (two or more test comparison)*
*Clues for one test: Bland-Altman, inter- and intra-rater agreement, Cohen's Kappa.*

**A SINGLE TEST: COMPARISON BETWEEN DIFFERENT TESTERS OR BETWEEN SEVERAL REPETITIONS OF THE TEST:**

**Yes- Code: Diagnostic agreement/reproducibility study:** A study where the repeatability (reproducibility) of a specific diagnostic test or procedure is evaluated by multiple observers (pathologists or others) or machines (image analysis, AI), or their combination. This may take the form of a test being repeated multiple times on the same cases at different time points or, more often, where different observers (e.g., a group of pathologists) evaluate and interpret/report the results of the test independently. The results of repeated testing are compared between the observers. Sometimes diagnostic agreement/reproducibility design may accompany diagnostic accuracy studies.

**TWO OR MORE TESTS: EVALUATION TO DETECT DIAGNOSIS (COMPARED WITH A REFERENCE/GOLD STANDARD):**

**Question 18: "Is the study primarily being conducted to validate the technical accuracy of a laboratory test, assay or diagnostic method?"**
i.e. records exploring the utility of pan-TRK immunohistochemistry (IHC) to detect NTRK fusions or comparing various 'field strengths' in a magnetic resonance.
If the study is comparing radiological, endoscopic or techniques of obtention of clinical samples, it would go to diagnostic test accuracy studies (i.e., mammograms vs histology)

**Yes- Code: Laboratory validation studies:** These studies **validate the performance of a laboratory test or assay** in a controlled laboratory environment (i.e., pathology laboratory), usually in cherry-picked cases/samples. **The focus is primarily on the technical aspects of the laboratory method** to ensure that it produces consistent and valid results when compared to the reference method. These studies are foundational for ensuring that a test is suitable for further clinical evaluation.

**Note: How to distinguish 'Diagnostic Test accuracy' Studies and Laboratory Test Validation studies?**

1. **Setting:** Diagnostic Test Accuracy Studies are often conducted in clinical settings with real patients, whereas Laboratory Test Validation Studies are conducted in controlled laboratory settings with often "selected" cases/patients.
2. **Focus:** Diagnostic Test Accuracy Studies focus on the clinical utility and application of the test in diagnosing patients, while Laboratory Test Validation Studies emphasize the technical precision and accuracy of the laboratory method itself.

3. **Outcome Measures:** While both types of studies evaluate sensitivity and specificity, Diagnostic Test Accuracy Studies also consider clinical scenarios and patient populations, while Laboratory Test Validation Studies focus more on technical consistency and accuracy in measuring the analyte.

**Question 19: "Is the study conducted to validate the clinical utility of an assay using comprehensive statistical analyses?"**

**Yes - Code: Diagnostic Test Accuracy-Study:** These studies evaluate the effectiveness and reliability of diagnostic tests in the clinical settings and focus on their ability to accurately detect the presence or absence of a particular disease or condition. **The focus here is on the clinical utility of the test**, i.e., assessing how well it helps to make accurate diagnostic decisions for a specific clinical question/situation. Therefore, the identification of selected patients and controls is mandatory as well as a comprehensive statistical analysis.

Key metrics used in these studies must include at least one of these options:

a) The area under the curve (AUC) of the receiver operating characteristic (ROC) curve.
b) Sensitivity, specificity, PPV, NPV: at least two of them **and** with 95% confidence interval (CI 95%)
c) Sensitivity, specificity, PPV, NPV: all of them without CI 95%

These studies are designed to quantify the performance of a test in a clinical setting and inform clinicians about the reliability of the test for making diagnostic decisions.

**No - Code: Cross-sectional (observational branch). Clarification:** The evaluation of the performance of a diagnostic test can be carried out based on data from analytical studies that do not have to be cross-sectional. But in any case, performance analysis always has a cross-sectional character. That is why they are derived towards that code.

**Question 20: "Did the investigator assign an exposure or intervention/screening strategy?"**
Intervention is a preventive or therapeutic regimen or a treatment to change outcomes on patient. It is included as an intervention the application of a diagnostic test.

***False Friend: "Intervention" There may be evaluation of interventions that are not assigned by the researcher*** *(e.g., searches for them in medical records). So, it is not an experimental study but an observational one.*
*Note: Do we exclude papers if a drug efficacy is assessed only? If they also evaluate prognostic or predictive factors, they should enter.*

**Yes – Go to Experimental Studies**

**No – Go to Observational Studies**

# EXPERIMENTAL STUDIES

**Question 21: "Are the intervention/control groups randomly allocated?"**
If the authors state in the abstract that the allocation was randomized, we will consider it as RCT.

**Yes - Code: Randomized Controlled Trial (RCT):** A clinical-epidemiological experiment in which subjects are randomly allocated into groups, usually called intervention/test and control groups, to receive or not to receive a preventive or a therapeutic procedure or intervention.

**No - Code: Non-randomized Controlled Trial:** Includes several types of study:

- Quasi-experimental: situation in which the investigator lacks full control over the allocation and/or timing of intervention but nonetheless conducts the study as if it were an experiment, allocating subjects to groups.
- Historical control: The control group are subjects who were studied before the main group receive the intervention.
- Experimental before-after studies: An approach in which the outcome is measured before and after an intervention that has been delivered to all participants.

# OBSERVATIONAL STUDIES

**Question 22: “Does the ti/ab state that the study is a Nested Case-control or Case-cohort?”**

**Nested Case-Control Study:** A type of case-control study in which cases and controls are drawn from a prospective cohort. Each time a case appears in the cohort, one or more controls are selected from among those who have not developed the outcome.

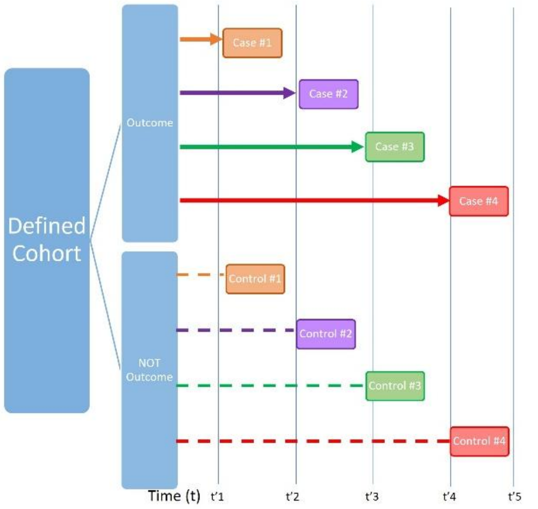


**Case-cohort:** The case cohort study includes all individuals from the cohort who have experienced the outcome of interest ("cases") and a sample of individuals randomly selected from the full cohort members observed at <u>baseline</u>.

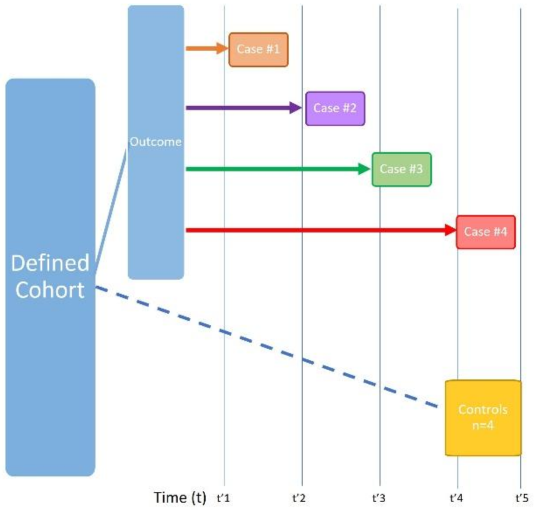


**Yes - Code: Cohort Study**
**No - Go to question 23**

**Question 23: “Is there follow-up of participants between exposure and outcome?”**
Follow-up is the observation over time of the participants, their exposures, the presence of outcomes or other variables of interest (i.e., survival, prognosis, time to event, etc.)
**Agreed keywords accepted as follow-up in abstract:** “survival” (despite not always when the abstract talks about survival, there is follow-up)

**Yes - Go to Question 24**
**No - Go to Question 26**

**Question 24: “Does it use RCT data?”** Studies may use cohorts of patients or data from randomised-controlled trials (see RCT definition). In this context, we mean studies *derived* from RCTs – work that is secondary or incidental to the original RCT aims. Thus, <u>RCT data is used for a new purpose</u>, but the study, regarding the new hypothesis of interest <u>is no longer an RCT, but an observational study</u> than utilises the information collected in a RCT.

*False friend: RCT: review the objective of the study to determine RCT or derived RCT?*

**Yes - Code: Observational Longitudinal Study RCT derived:** The information and/or samples collected in an RCT are used to test a different hypothesis (i.e. the prognostic value of a biomarker). Patients were not randomized according to the new objective, so it is an observational study taking advantage of the systematic collection of data and the quality control measures usually included in RCTs.

**Question 25: Is there a quality formal survival-time to event comparison (incidence, recurrence, death...) AND (n>100 if prospective or n>500 if retrospective)?**

Some studies assess survival or prognosis but do not perform high quality follow-up and are therefore not considered cohort studies. Quality follow up is considered if there is control of each patient included at the baseline (considering losses during follow-up) which makes it possible to calculate with certainty: incidence rate ratio, Poisson regression, Kaplan Maier survival curve, Hazard ratio (HR), Cox regression, attributable risk, Log Rank test, predictive value for survival, relative risk (RR), comparison of median survival time [or 50% survival], comparison of overall survival (OS), comparison of disease free survival (DFS).

*Agreed key word for "yes": (association + survival) = quality follow-up*
*Clues for no: Odds ratio*

**Yes - Code: Cohort Study:** Study design in which the investigator observes participants (free of the event that is going to be evaluated) who are exposed and unexposed and follows them up to see if they develop the disease or event of interest. In a cohort study, the investigator observes and does not assign the participants' exposure status as in the experimental design. They could be prospective or retrospective (conducted by reconstructing data about persons at a time or times in the past). If the cohort does not reach at least 100 participants, it will be coded as "Small Cohorts & Other Longitudinal Studies".

Cohort criteria:

- Quality measures AND
- Sample size (if prospective: n>100, if retrospective: n>500) -if not clearly prospective by abstract, consider as retrospective-.

**No - Code: Small Cohorts & Other Longitudinal Studies:** This category includes:

- Observational before-after Study: An approach in which the outcome is measured before and after an exposure has occurred to all participants. In contrast to the experimental ones, the exposure is not defined by the researcher.
- Low-quality follow up studies**:** other longitudinal studies that do not meet the criteria for cohorts, they may compute percentage of survival patients or other index typical of cohort studies, but without controlling loses of follow-up and/or using survival analytical methods.

**Question 26: "Does the study compare affected with "healthy" tissue from the same individual?"**
Requirement for "yes": The compared tissue must not be peritumoral or adjacent tissue (same slide will be considered always adjacent tissue)
**If the study doesn't meet this criterion, it will be coded as cross-sectional.**
*False friend: "healthy tissue" or "normal tissue" don't necessary meet the requirements.*

**Yes - Code: Self-matched Case-Control Study**: in this design, each participant (case) serves as its own matched control. I.e., when the tumour tissue from a cancer case is compared with the corresponding "healthy" tissue from the same patient to assess the association between some characteristic/feature/exposure with the outcome (disease or cancer subtype).

**Question 27: "Is there a selection of defined cases and a set of comparable controls?"**
*Clues for no*
*Comparisons of histological, immunohistochemical, genetic, etc. profiles between groups of tumours are cross-sectional.*
*Comparisons: Tumour vs somatic DNA: cross-sectional*
*False friend "control" Some studies uses the word "control" but they are not actually "comparable control"*
*Clues for yes:*
*Matched control*
*Comparison Tumour vs Germline DNA: case control (this DNA already existed before the cancer appeared and is*

**Yes - Code: Classic Case-Control Study:** Study design in which the investigator selects participants with the outcome (cancer type or subtype) (cases) and a comparison group without that particular outcome (controls) to study the association between previous exposures or previous characteristics with the outcome. Cases and controls are very often matched by age, sex, or other variables.

**No/Unclear - Code: Cross-Sectional Study:** Study in which the outcome and other variables are determined in each participant of the study at one particular time. It can refer to a period, however observed photographically (without evolution).

There is no formal selection of controls, this means that firstly the participants are recruited and only after that, they are classified depending on their diagnosis into the comparison groups (sometimes referred as cases and controls). **Comparisons of histological, immunohistochemical, genetic, etc. profiles between groups of tumours are included here (exception: in this case it is considered cross-sectional even with a formal selection of controls)**.

# References


1. Porta MS, Greenland S, Hernán M, Silva I dos S, Last JM, International Epidemiological Association, editors. A dictionary of epidemiology. Sixth edition. Oxford: Oxford University Press; 2014. 343 p.
2. Silman AJ, Macfarlane GJ, Macfarlane TV. Epidemiological studies: a practical guide. Third edition. Cambridge: Cambridge University Press; 2019. 278 p.
3. NICE [Internet]. NICE; [cited 2023 Sep 21]. Glossary. Available from: https://www.nice.org.uk/glossary?letter=b
4. Celentano DD, Szklo M, editors. Gordis, epidemiología. 6a edición. Barcelona, España: Elsevier; 2020.
5. Damián Moreno J, Royo Bordonada MÁ, editors. Manual de Método epidemiológico.
6. Piédrola Gil G. Medicina preventiva y salud pública. 12ª ed. Barcelona: Elsevier Masson; 2016.
7. Método epidemiológico. Madrid: Escuela Nacional de Sanidad; Instituto de Salud Carlos III; 2009.
8. Argimon Pallás JMaría. Métodos de investigación clínica y epidemiológica. 3a. ed. Madrid: Elsevier; 2005.
9. National Institute for Health and Care Excellence. Methods for the development of NICE public health guidance [Internet]. 3rd ed. NICE; 2012 [cited 2023 Jul 13]. 286 p. Available from: www.nice.org.uk/process/pmg4
10. National Clinical Effectiveness Committee Ireland. Standards for Clinical Practice Guidance [Internet]. Dublin: NCEC; 2015. 31 p. Available from: https://www.nmbi.ie/NMBI/media/NMBI/Forms/standards-for-clinical-practice-guidance-ncec.pdf
11. Centre for Evidence-Based Medicine. Study designs [Internet]. [cited 2023 Jul 13]. Available from: https://www.cebm.ox.ac.uk/resources/ebm-tools/study-designs
12. O'Brien KM, Lawrence KG, Keil AP. The Case for Case–Cohort: An Applied Epidemiologist's Guide to Reframing Case–Cohort Studies to Improve Usability and Flexibility. Epidemiology. 2022 May;33(3):354–61.
13. Guyatt G, Oxman AD, Akl EA, Kunz R, Vist G, Brozek J, et al. GRADE guidelines: 1. Introduction—GRADE evidence profiles and summary of findings tables. Journal of Clinical Epidemiology. 2011 Apr 1;64(4):383–94.
14. Peng B, Amos CI. Forward-time simulation of realistic samples for genome-wide association studies. BMC Bioinformatics. 2010 Sep 1; 11:442.
15. Indave BI, Colling R, Campbell F, Tan PH, Cree IA. Evidence-levels in pathology for informing the *WHO classification of tumours* . Histopathology. 2022 Oct;81(4):420–5.
16. Higgins J, James T, Chandler J, Cumpston M, Li T, Page M, et al., editors. Cochrane Handbook for Systematic Reviews of Interventions Version 6.3. (updated February 2022) [Internet]. Cochrane; 2022. Available from: https://training.cochrane.org/handbook/current
17. Computer Simulation - MeSH - NCBI [Internet]. [cited 2023 Jul 13]. Available from: https://www.ncbi.nlm.nih.gov/mesh/68003198
18. Animal Experimentation - MeSH - NCBI [Internet]. [cited 2023 Jul 13]. Available from: https://www.ncbi.nlm.nih.gov/mesh/68032761
